# The effectiveness of Ghana’s antibiotic control policy framework: an analysis using the Anderson governance framework

**DOI:** 10.1080/20523211.2026.2670691

**Published:** 2026-05-20

**Authors:** Radolf Ansbert Nortey, Irene Akwo Kretchy, Mercy Naa Aduele Opare-Addo, Kwame Ohene Buabeng

**Affiliations:** a Department of Pharmacy Practice, Faculty of Pharmacy and Pharmaceutical Sciences, Private Mail Bag, University Post Office, Kwame Nkrumah University of Science and Technology, Kumasi, Ghana; b Department of Pharmacy Practice and Clinical Pharmacy, School of Pharmacy, University of Ghana, Legon, Ghana

**Keywords:** Antibiotic, policy, antimicrobial resistance, controlled medicines, action-plan, stakeholders

## Abstract

**Background:**

The burden of antimicrobial resistance is rapidly increasing, with the highest rates anticipated in Sub-Saharan Africa. Most countries around the world have expressed political commitment to strengthen antibiotic control and have adopted corresponding legislative policies. There are also repeated calls for more rigorous antibiotic controls and a coordination framework akin to the international system for controlled medicines. Ghana has instituted a policy on antimicrobial use and resistance, as well as a now-expired National Action Plan (NAP) on Antimicrobial Resistance (AMR). While there is growing support for stricter antibiotic control, concerns remain regarding the suitability of such interventions in local settings. This study examined the effectiveness of Ghana’s antibiotic control policies through the lens of the Anderson Governance framework.

**Methods:**

The study employed semi-structured interviews with purposively selected stakeholders from the antibiotic control landscape in Ghana. The interviews explored the effectiveness of Ghana’s antibiotic control policy and the NAP on AMR within the structural domains such as policy design, implementation tools, monitoring, and evaluation. The emerging data was coded and analysed thematically using Atlas.ti, guided by the Anderson Governance Framework and the COREQ guidelines.

**Results:**

Seventeen (17) stakeholders participated in the study. Participants described several challenges, including fragmented coordination, limited availability of data, insufficient stakeholder inclusivity, barriers to access and minimal use of digital tools. Although participants expressed a need for improved AMR surveillance, several cautioned that adopting a similar system to the international drug control framework could be detrimental to antibiotic access.

**Conclusion:**

The study highlights significant gaps within Ghana’s antibiotic control policy framework, particularly in coordination, the integration of emerging digital technologies, and the inclusion of frontline implementers’ perspectives. While restrictive control models may compromise access to essential antimicrobials, integrating selected digital components of such systems may offer useful insights for improving AMR surveillance.

## Background

Antibiotics remain a critical bedrock of medicine and the rapid emergence of antimicrobial resistance (AMR) threatens the viability of national health systems and the global economy (Murray et al., 2022). Due to increasing resistance, doctors are often using ‘last-resort’ or ‘last-line’ antibiotics that are more costly and less accessible in low to middle income countries (World Health Organization, 2016). In May 2015, the 68th World Health Assembly made a political commitment to support the implementation of National Action Plans (NAP) on antimicrobial use and endorsed a global strategy to address the issue of emerging resistance (Charani et al., 2023; World Health Organization, 2016). The purpose of the NAP is to enhance public awareness on AMR, strengthen surveillance systems, improve research, reduce infection incidence, and increase investment in relevant applicable interventions (Charani et al., 2023; World Health Organization, 2016).

However, the commitment to NAPs at the local level holds little to no value when these plans lack effective implementation or can be described as ‘ghost policies’ (Charani et al., 2023). The existing global coordination for antibiotic control is weak and a recent analysis of 78 NAPs from different global regions unearths a widely disconnected process for data collection and antibiotic surveillance (Charani et al., 2023; Hoffman et al., 2015; Willemsen et al., 2022). Various reports have gone on to discuss the suitability of a more rigorous control for antibiotics and a coordination framework similar to the international system for controlled medicines (Hoffman et al., 2015; Storehagen et al., 2018). Controlled medicines are drugs or chemicals whose manufacture, purchase, distribution is legally regulated due to the strong potential for diversion into illicit use (Burke-Shyne et al., 2017). They include narcotic substances such as morphine or codeine, psychotropic substances like diazepam and precursors (International Narcotics Control Board (INCB), 2019, 2024).

The current drug control system for controlled medicines is prescribed under the United Nations 1961 Single Convention on Narcotic Drugs and other related legal instruments (United Nations, 1961). The International Narcotics Control Board (INCB) exerts regulatory oversight on its implementation and outlines detailed specifications for the reporting, data collection, tracking, and monitoring to ensure their safe legitimate use (United Nations, 1961). For countries to effectively assess their capacity for data collection and surveillance, a situational analysis of existing system capabilities is essential (Charani et al., 2023). This assessment should be clearly integrated into their National Action Plans (NAPs) on Antimicrobial Resistance (AMR) (Charani et al., 2023; Murray et al., 2022). Ghana launched a 5-year National Action Plan on Antimicrobial Resistance (NAP) in 2018, intended to run until 2021. However, to date, there has been no formal review of its outcomes or an update outlining the strategy and projections for the next phase (Jimah & Ogunseitan, 2020).

There is increasing evidence to suggest a continual increase in antimicrobial misuse and the absence of an effective control system (Jimah & Ogunseitan, 2020; Nortey et al., 2022, 2026). This study seeks to assess the effectiveness of the NAP in Ghana vis-à-vis the need for an enhanced control system from the perspectives of stakeholders using the Anderson Governance framework.

The Anderson framework is a health system conceptual framework utilised in the development and assessment of national action plans on AMR (Anderson et al., 2019). It was originally designed as cyclical process with three main thematic areas: Policy Design, Implementation Tools, and Monitoring and Evaluation (Anderson et al., 2019). Each of these areas addresses key components for effective policy development and implementation, ensuring a comprehensive approach to tackling antimicrobial resistance (AMR). The cyclical conceptualisation reflects the dynamic nature of AMR and the continuing need for process modification and adaptation (Ahmed et al., 2022; Anderson et al., 2019).

## Method

### Research design

The study employed an exploratory qualitative approach to evaluate Ghana’s AMR Policy and the National Action Plan (NAP) on AMR (2017–2021) based on perspectives from key stakeholder groups in antibiotic control. The design was guided by the Anderson Governance framework and the COREQ guidelines, enabling an in-depth exploration of stakeholder perspectives, policy strengths, gaps, and areas for improvement. Because stakeholder insights spanned multiple domains of the Anderson Governance framework, participants were not assigned to specific categories. The analysis examined the policy’s alignment with global frameworks, its effectiveness in addressing Ghana’s AMR challenges, and the status of implementation.

### Study population and sampling

The study population consisted of seventeen (17) key stakeholders purposively selected from the antibiotic policy and control landscape in Ghana (Table 1). These selected individuals played key roles in the development, implementation, and evaluation of Ghana’s AMR Policy and NAP. Participants included members of the National AMR Committee, alongside representatives from the Ministry of Health, Ghana Health Service, Ministry of Agriculture, Food and Drugs Authority (FDA), Veterinary Services, Pharmaceutical Society of Ghana (PSGh), Academia, industry representatives and researchers. The sample size determination was informed by the principle of data saturation and the quality of the data obtained, where gathering new data no longer unearthed any new insights (Vasileiou et al., 2018).

**Table 1.** Stakeholder institutions involved in key informant interviews.

Institution/Agency	Number of respondents
Food and Drugs Authority (FDA)	3
Pharmaceutical Society of Ghana (PSGh)	1
Ministry of Health	2
Ministry of Food and Agriculture (AMR Reference Lab for Animal Health)	1
Ghana Health Service	1
University of Ghana Medical Centre (UGMC) – Quaternary Care	2
University of Ghana	1
Pharmacy Council	1
Community Practice Pharmacists Association of Ghana (CPPA)	1
Pharmaceutical Manufacturers Association of Ghana	1
Innovator Pharmaceutical Companies (Antibiotics)	1
Pharmaceutical Importers & Wholesalers Association of Ghana (PIWAG)	1
Ghana College of Pharmacists	1

### Data collection

**Figure 1.**
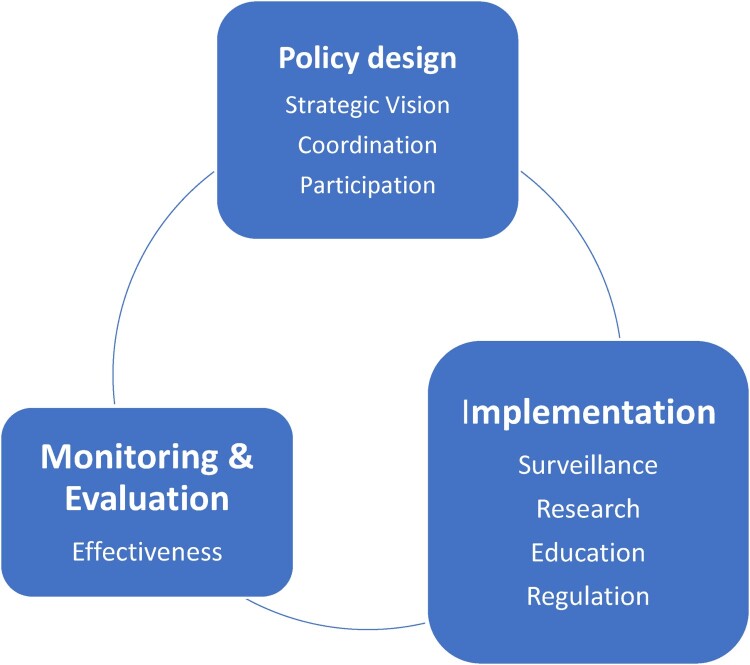
Framework for assessment of National Action Plans. Source: Amended from Anderson et al. (2019). This figure illustrates the three core domains assessed in the study: policy design, implementation, and monitoring and evaluation, along with their associated subcomponents.

Data collection for the study was carried out using semi-structured interviews with key informants. This method enabled broader flexibility while ensuring the exploration of predefined themes (Adeoye-Olatunde & Olenik, 2021; DeJonckheere & Vaughn, 2019). A well-designed interview guide based on the Anderson governance framework for AMR policies provided a structural framework for the interviews (Figure 1). The interview guide consisted of open-ended questions aligned with the key domains of the Anderson Governance framework: strategic vision, coordination, implementation tools, monitoring and evaluation, and sustainability (Table 2). The questions further examined stakeholder insights into the functionality of Ghana’s National Action Plan (NAP) on AMR. For instance, discussions on strategic vision explored how stakeholders perceived the relevance of the NAP to national AMR priorities and its alignment with global standards. This interview guide was piloted with four (4) colleague pharmacists in Accra, Ghana to ensure the questions could effectively elicit the right information. Following the pilot interviews, no questions required refinement or changes. All the interviews were conducted in English by the first author (RAN), who is a male pharmacist and a PhD candidate in Social Pharmacy with experience in qualitative and quantitative research.

**Table 2.** Interview guide based on the Anderson governance framework.

Construct	Item from interview guide
Strategic vision and coordination	1. Was your organisation involved in developing Ghana’s AMR NAP? If so, what was its role, and how would you assess the plan’s strategic vision? 2. How effective is collaboration between the human, animal, and environmental health sectors in addressing AMR? What challenges exist in fostering intersectoral coordination?
Stakeholder engagement	3. How would you evaluate the level of stakeholder participation in the design and implementation of the AMR NAP? 4. In our study, almost all the pharmacists had no idea about the existence of a policy/ action plan. Any comments about this observation? 5. What role do you see pharmacists playing in guiding antibiotic use, and how can their practices be regulated or enhanced to discourage OTC antibiotic purchases? 6. What policies or training could empower providers to promote responsible antibiotic use?
Medicines regulation and public knowledge	7. What is your assessment of public knowledge about the risks of antibiotic misuse, such as resistance and side effects? 8. How well are current regulations on antibiotic sales enforced, and what are the main challenges in this regard? 9. Controlled medicines such as morphine and pethidine have a more rigorous control framework with tracking and data collection systems. Do you think antibiotics need to be reclassified to enable stronger control systems?
Research and data gaps	10. How well does the current AMR NAP reflect existing research on antibiotic use and resistance in Ghana? 11. In our study, we identified gaps in data on antibiotic consumption or distribution. Are there any ongoing efforts to close these gaps or increase data collection on antibiotic use?
Policy effectiveness and recommendations	12. Has the AMR NAP been evaluated for its impact? What metrics or indicators are used to assess its effectiveness? 13. What are your recommendations for improving future policies or action plans on AMR?

Participants were recruited from stakeholder institutions referenced in the AMR Policy and NAP. Prospective participants were contacted via email or phone to arrange an appointment. Four (4) potential participants declined due to time constraints but referred the researcher to a suitable colleague within the same department. Most participants were pharmacists, and the researcher’s professional background facilitated rapport, engagement, and willingness to participate.

Nine (9) stakeholders agreed to face-to-face interviews, held in their respective offices, while the remaining eight (8) preferred virtual meetings, conducted via Microsoft Teams. All interviews were conducted in settings that ensured privacy and minimal interruptions. Each participant was interviewed only once, and no follow-up or repeat interviews were conducted. The interviews lasted between 20 and 30 min and were audio-recorded with the prior consent of participants.

### Data analysis

The interview data were transcribed verbatim and analysed (by RAN and IAK) using thematic analysis to identify recurring themes, gaps, and stakeholder perspectives. The main thematic areas of the interviews were synthesised and categorised using a stepwise process which involved data familiarisation, coding, and theme identification (Braun & Clarke, 2021; Clarke & Braun, 2013).

The analysts first acquainted themselves with the transcripts and developed a broad thematic template based on the interview questions. The Anderson Governance Framework served as a guiding analytical tool with its key governance domains (e.g. policy design, implementation tools, monitoring, and evaluation) forming the main thematic categories (Anderson et al., 2019). A codebook was subsequently developed to capture the identified themes which were derived from common patterns across participants’ responses. The research team discussed the emerging themes in relation to the Anderson Governance framework until they arrived at a consensus on the final themes/subthemes with supporting quotes from the interviewees.

Data was coded using Atlas.ti qualitative analysis software, allowing for systematic identification of themes and sub-themes. To enhance the credibility of the findings, a member-checking process was adopted. Preliminary findings were shared with select participants for validation, ensuring that the analysis accurately reflected stakeholder insights and avoided misinterpretation. The data analysis was guided by the COREQ checklist (Online Resource 1) to ensure the credibility, transferability, and originality of the study (de Jong et al., 2021; Lincoln & Guba, 1986).

### Ethical consideration

The Ghana Health Service Ethics Committee approved this study under the registration code GHS-ERC: 008/05/21. To guarantee participant confidentiality, no personal identifiable information was collected or linked to any of the recordings. In accordance with ethical protocols, all the interviewees were briefed on the purpose of the research and signed a consent form prior to participation. Transparency was a priority, and no participants were coerced, intimidated, or financially induced to participate. Participants retained the right to decline participation or withdraw at any point without consequence.

## Results

This study findings presents the key findings from an analysis of Ghana’s National Action Plan (NAP) and antibiotic control policies, using an adapted version of the Anderson’s governance framework. The results are structured around three core governance pillars: Policy Design, Implementation Tools, and Monitoring & Evaluation, which serve as the main thematic areas for organising the analysis.

**Figure 2.**
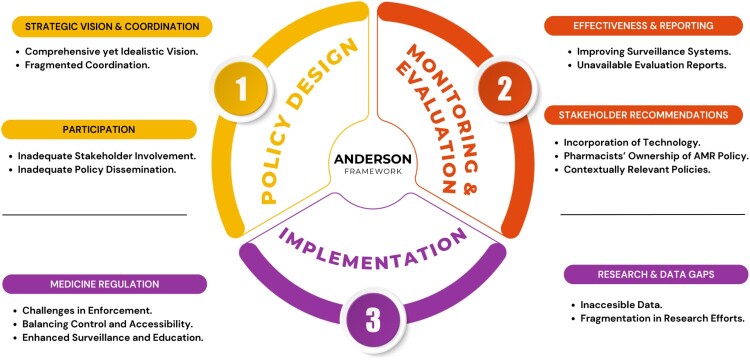
Stakeholders’ responses regarding Ghana’s AMR response in the Anderson framework. This figure summarises the coded interview themes classified under the domains of the Anderson Governance Framework, providing an overview of Ghana’s AMR response and control framework from the perspectives of key stakeholders.

Figure 2 summarises how participants’ responses were mapped onto the domains of the Anderson Governance Framework. After coding the interviews, each theme was classified under one of the six governance domains, providing a structured overview of stakeholders’ perceptions of Ghana’s AMR response and control framework. Each identified theme provides valuable insights into the country’s policy landscape and highlights critical areas for strengthening antimicrobial stewardship and governance. The quotations from respondents are italicised and are referenced by the interview number and the core mandate of the stakeholder institution.

### Policy design

#### Strategic vision and coordination

##### Comprehensive yet idealistic vision

Participants viewed the National Action Plan (NAP) on AMR as comprehensive but yet more idealistic than realistic.

The strategic plan on paper is too ambitious, it's very exhaustive and very strong … but it's only excellent on paper. (Interview #2, Regulatory agency)

The vision is robust and well-articulated, but the real issues come when we attempt to implement it simultaneously (Interview #4, Professional Association)

##### Fragmented coordination

Many participants reported significant challenges in cross-sectoral collaboration, leading to fragmented coordination.

In terms of collaboration between the health sectors, that is the human, animal, and environment, the short answer is that it has not been effective. We all talk about the One Health approach … but practically, and I believe it's as a result of logistical constraints, it's been impossible to collaborate. Data sharing is a challenge! (Interview #7, Policymaker)

### Policy design

#### Stakeholder engagement

##### Inadequate stakeholder involvement

Respondents reported varied levels of engagement among key actors during the design of the AMR policy and noted weaker stakeholder participation during its implementation or evaluation.

We need to look at the implementation of the existing policy, and we need to involve the stakeholders, not look at it from a top-down approach, but from a bottom-up approach, so that the stakeholders feel like they are part of contributing to the implementation of the policy. (Interview #13, Pharmacy Practice)

##### Inadequate policy dissemination

Some participants highlighted an uneven dissemination of the AMR policy with some level of disparity between pharmacists in the public and private sectors.

In all honesty, I had no idea about the NAP until I joined the government agency. So I think more awareness and education should be conducted because I don’t think it’s that popular in the pharmaceutical space (Interview #3, Regulator)

### Implementation

#### Medicine regulation

##### Challenges in enforcement

Participants noted challenges in the regulatory control of antibiotics.

The policies and regulations are there, but monitoring seems to be almost non-existent. Regulators clearly don’t have adequate market oversight or capacity. (Interview #5, Policymaker)

##### Balancing control and accessibility

Participants expressed concerns about the consequences of imposing excessive restrictions on antibiotic access.

If we make antibiotics very difficult to access, we are going to try to solve a problem and create another problem. Simple ailments that can be simply treated at the community level would escalate into outbreaks and stuff like that. (Interview #17, Pharmacy Training)

I think they should be classified, but not as morphine and other opioids. So, I think they should be reclassified and regulated. That would be the best for antimicrobials, it’s a crisis! (Interview #13, Pharmacy Practice)

##### Enhanced surveillance and education

Participants acknowledged notable progress in AMR control in Ghana, especially increased awareness and enforcement in urban areas.

Current campaigns and regulations have been somewhat effective, especially in urban areas. Now we have community pharmacies that, when you walk in, they refuse to sell antibiotics to you without a prescription. (Interview #3, Regulator)

### Implementation

#### Research and data gaps

##### Inaccessible data

Respondents described challenges related to incomplete AMR data coverage and lack of integration across health facilities.

There is a lot of AMR data at the various teaching hospitals, but what about the small clinics and pharmacies that freely dispense antibiotics? These are left out, making the data incomplete. (Interview #9, Infectious Disease Management)

##### Fragmentation in research efforts

Participants noted the absence of comprehensive and harmonised data collection on AMR.

There is a huge disconnect between researchers and policymakers. If researchers don’t even know about the national AMR strategy, how can they contribute effectively? (Interview #6, Policymaker)

### Monitoring & evaluation

#### Effectiveness & reporting

##### Improving surveillance systems

The stakeholders reported recent initiatives aimed at improving antibiotic data collection and utilisation especially in the area of digitalisation.

Some applications have been developed that seek to solve this problem. The data is available but it's about making the data or reorganizing the data in a form that is analyzable meaningfully to inform decisions. (Interview #5, Policymaker)

##### Unavailable evaluation reports

Most stakeholders highlighted concerns about a lack of communication regarding NAP evaluations and the absence of efficient reporting systems.

So, I don't remember any form of monitoring and evaluation on the NAP. It is possible it may have happened without my knowledge. (Interview #7, Policymaker)

### Monitoring & evaluation

#### Stakeholder recommendations

##### Incorporation of technology

Stakeholders highlighted the importance of integrating modern advancements and technology into future AMR policies.

I think we should look at what it was, what we have or what we had and see if, with the knowledge that we have today, I mean, looking at even electronic pharmacy services and see how we can incorporate that into the plan. (Interview #1, Regulator)

##### Contextually relevant policies

Participants indicated a need for policies to be tailored to the specific socio-cultural and economic conditions in Ghana.

For policies to be truly effective, they must incorporate local knowledge, community perspectives, and grassroots-level interventions. (Interview #16, Industry Expert)

##### Pharmacists’ ownership of AMR policy

The stakeholders described the involvement of pharmacists as a critical role in antibiotic stewardship.

Pharmacists are at the forefront of ensuring that individuals do not misuse antibiotics, which is a significant factor contributing to antimicrobial resistance. (Interview #17, Pharmacy Training)

## Discussion

Stakeholders indicated that the policy framework for AMR in Ghana presented a comprehensive strategic vision, but one that is overly ambitious and impractical within the current landscape of antibiotic access and use. Using the Anderson Governance framework, this reflects weaknesses in the policy design domain, suggesting that the strategic vision does not adequately account for context-specific challenges such as financing, personnel, resource constraints, and political barriers. Consistent with previous studies, such misalignment between policy design and implementation capacity often results in limited success and has been described in the literature as ‘ghost policies’ (Charani et al., 2023; Nortey et al., 2026).

Prescription-only access for antibiotics appears to be an impractical situation for many LMICs, where medicine supply units are commonly manned by non-pharmacists (Afari-Asiedu, Oppong, et al., 2020; Nguyen et al., 2019; Nortey et al., 2022, 2026). This reinforces the need for a more context-specific approach to the development of national AMR policies and action plans. Perspectives from respondents also suggest a skewed policy recognition of the role of pharmacy practitioners and, more broadly, an inadequate involvement of all relevant stakeholders. Viewed through the Anderson Governance Framework, this reflects weaknesses in the stakeholder participation domain and highlights potential challenges in achieving coordinated and effective enforcement of AMR policies.

Regarding implementation, while participants consistently recognised the importance of regulating antibiotics to prevent antimicrobial resistance, they cautioned against extreme control measures. Specifically, they noted that adopting a similar system to the international drug control framework for controlled medicines would adversely inhibit access, present implementation challenges, and incur higher costs, concerns that have also been reported in other settings (Storehagen et al., 2018).

At the same time, participants acknowledged that maintaining the status quo also implied endorsing the irrational use of antibiotics, a challenge well documented in Ghana and similar contexts (Afari-Asiedu et al., 2018; Torres et al., 2019; Yevutsey et al., 2017).

Thus, the findings suggest a need for strengthened regulatory oversight, but not necessarily through a system akin to the narcotic drug control apparatus. Instead, the potential value of selectively adapting specific elements was highlighted. For instance, the digital tools offer transferable lessons for antibiotic stewardship (International Narcotics Control Board, 2025). The documented global success of these platforms highlights the feasibility of digitalisation in tracking medicines and enhancing antibiotic surveillance across Ghana (Charani et al., 2023; Eisenmann et al., 2024; Matthess & Kunkel, 2020; Rawson et al., 2024).

Kenya recently recorded significant progress with its One Health AMR Surveillance System (OHAMRS) which has been pivotal in understanding AMR prevalence and patterns across multiple sectors (Chuchu et al., 2024). The value of digital systems in enabling real-time monitoring and process simplification highlights the need for strategies to maximise the use of technology (Charani et al., 2023; Chuchu et al., 2024). Policy actors should not view technology adoption as limited to large, expensive software systems; it also includes the everyday use of simple tools like Microsoft Office, point-of-sale applications, and other practical digital solutions.

The study findings also indicate an over-prioritisation of antibiotic use in public hospitals and a disparity of control between urban and rural sectors. Consistent with existing literature, antibiotic stewardship interventions are primarily focused on clinical settings where prescribing behaviour can be monitored vis-à-vis laboratory evidence of infection (Torres et al., 2019; Wilkinson et al., 2018). Community pharmacies are marginally affected or included in targeted initiatives on rational antibiotic use (Sulis & Gandra, 2021; Wilkinson et al., 2018). The policy actors in Ghana attributed this situation to uncoordinated retail systems and challenges in regulatory enforcement. Beyond the call for more education as a solution, there is also a need for a synchronised antibiotic regulatory framework with a feedback mechanism for monitoring (Khare et al., 2019; Sulis & Gandra, 2021).

In the rural communities in Ghana, residents were unable to differentiate antibiotics from other common OTC medicines such as paracetamol (Afari-Asiedu, Hulscher, et al., 2020; Do et al., 2021). This knowledge gap negates the maximum effect of AMR communication campaigns especially in rural settings (Do et al., 2021). The findings of this study corroborate this observation, suggesting that AMR sensitisation efforts are more effective in urban communities and that communication should be tailored to different audiences (Charani et al., 2023; Travasso, 2016).

Quite a number of studies have called for specific culturally tailored approaches to the use of antibiotics outside hospital settings (Afari-Asiedu, Hulscher, et al., 2020; Do et al., 2021; Nortey et al., 2026). However, these recommendations remain listed on paper and are not reflected in the NAP for antibiotic control in Ghana (National Action Plan (NAP) for Antimicrobial Use and Resistance in Ghana, 2017). Participants in this study characterised the situation as a ‘disconnect between researchers and policymakers’. This highlights the need for a more enhanced synthesis of the available data to better inform policy makers in the review and update of the NAP.

## Implications for policy and practice

### Targeted research

Stakeholders in academia and research need to invest in comprehensive synthesis and meta-analyses of available local studies on non-prescription antibiotic use, to provide policy makers with the necessary evidence-based insights to inform effective policy. Similarly, the Ministry of Health (MOH) is to ensure the next review of the NAP is informed not just by anecdotal evidence and international best practices, but also by a thorough aggregation of local data and context-specific recommendations to guide decision-making.

### Clarity in agency stewardship responsibilities

The Ministry of Health (MOH) needs to consider clarifying the roles of stakeholder agencies in antibiotic control especially in data gathering and surveillance. Most MOH agencies declare a shared mandate but are unable to outline specific roles. In line with the INCB’s mandatory reporting requirements for controlled medicines, the FDA and Pharmacy Council (PC) should be held strictly accountable by the Ministry of Health (MOH).

### Stakeholder representation

The Ministry of Health and Pharmaceutical Society of Ghana (PSGh) must ensure a more inclusive approach in stakeholder consultations.

### Digitalised surveillance

MOH must ensure that subsequent editions of the NAP include a component of digitalisation in surveillance. The FDA and PC must also liaise with partners in academia to encourage more local implementation research on the incorporation of digital tools in antibiotic control.

### Transparency in implementation

To promote greater inclusion and accountability, the Interagency Committee on Antibiotic Control should consider publishing quarterly reports on the Ministry of Health’s website.

## Strengths & limitations of the study

The study did not include a comprehensive document analysis to systematically identify policy and implementation gaps, instead it relied solely on stakeholder opinions which are susceptible to subjective bias from both the respondents and researchers. However, rigorous, structured, and comprehensive processes were employed during the study design, data collection and analysis to minimise this potential bias (Bergelson et al., 2022).

Additionally, the findings are based on self-reported behaviours, which may have been influenced by social desirability bias. Given the sensitivity of the subject, especially when evaluating initiatives for which respondents were directly involved or accountable, some participants may have provided responses aimed at protecting their personal or institutional image (Grimm, 2010). Nonetheless, ensuring data anonymity helped mitigate this issue by making participants feel more comfortable sharing their views openly (K. M. Mwita, 2022).

Furthermore, the sample primarily comprised institutional and policy-level stakeholders, which may have influenced the range of perspectives and resulted in limited representation from frontline prescribers, community pharmacists, and informal providers. Future research should integrate these groups more directly to provide a more comprehensive understanding of antibiotic access, stewardship practices, and implementation challenges across community settings.

## Conclusion

There is considerable evidence of antibiotic misuse in Ghanaian community settings, yet the most recent NAP on AMR does not strongly reflect community-level interventions nor adequately incorporate the perspectives of frontline stakeholders. This study identified significant gaps across policy design, implementation, and monitoring and evaluation within Ghana’s antibiotic control landscape. Stakeholders acknowledged the strength of the existing AMR policy framework but noted that limited resources, coordination challenges, and insufficient stakeholder participation continue to hinder effective implementation. Notably, the international drug control system offers valuable lessons for effective antibiotic surveillance and data collection through its digital tracking components.
